# “*I Feel Therefore I Decide*”: Effect of Negative Emotions on Temporal Discounting and Probability Discounting

**DOI:** 10.3390/brainsci11111407

**Published:** 2021-10-25

**Authors:** Cinzia Calluso, Maria Giovanna Devetag, Carmela Donato

**Affiliations:** Department of Business and Management, Luiss University, Viale Romania 32, 00192 Roma, Italy; mdevetag@luiss.it (M.G.D.); donatoc@luiss.it (C.D.)

**Keywords:** negative emotions, intertemporal choice, risky choice, emotion regulation, self-regulation

## Abstract

Temporal and probability discounting are considered two fundamental constructs in economic science, as they are associated with phenomena with major societal impact and a variety of sub-optimal behaviors and clinical conditions. Although it is well known that positive and negative affective states bear important cognitive/behavioral consequences, the effect of emotional experiences on decision-making remains unclear due to the existence of many conflicting results. Inspired by the need to understand if and to what extent the current COVID-19 pandemic has determined changes in our decision-making processes by means of the unusual, prolonged experience of negative feelings, in this study we investigate the effect of anger, fear, sadness, physical and moral disgust on intertemporal and risky choices. Results show that all emotions significantly increase subjects’ preferences for immediate rewards over delayed ones, and for risky rewards over certain ones, in comparison to a “neutral emotion” condition, although the magnitude of the effect differs across emotions. In particular, we observed a more pronounced effect in the case of sadness and moral disgust. These findings contribute to the literature on emotions and decision-making by offering an alternative explanation to the traditional motivational appraisal theories. Specifically, we propose that the increased preference for immediate gratification and risky outcomes serves as a mechanism of self-reward aimed at down-regulating negative feelings and restore the individual’s “emotional balance”.

## 1. Introduction

After more than one year into the COVID-19 pandemic, experts and policymakers across the world are expressing increasing concern about the psychological and emotional toll that the spread of the coronavirus disease and the consequent restrictions to mobility have imposed on populations in the most heavily affected countries. Several reports published since the pandemic outbreak in March 2020 have signaled an alarming increase in rates of diagnosed mental disorders such as depression, anxiety, and Post-Traumatic Stress Disorder in response to health-related concerns, prolonged lack of sociality and high uncertainty about the future.

The Pew Research Center reported fear, panic, anger, sadness, health anxiety, and economic/financial anxiety as the most highly prevailing emotions [1]. Not only, in fact, the rapid surge of the pandemic has caused preoccupation about the disease’s short- and long-term health consequences, especially among the elderly, but the resulting economic downturn accompanied by the massive loss of jobs and the social isolation caused by lockdowns, mandatory mask wearing, and prolonged interruption of many leisure activities has triggered a whole spectrum of negative emotions associated with loneliness, economic uncertainty and financial insecurity The Pew Research Center reported evidence concerning the US population according to which at least one-third of Americans experienced high levels of psychological distress during the coronavirus outbreak. Overall, 36% of Americans said the coronavirus outbreak represented a major threat to day-to-day life in their community, according to a related survey [1]. Almost one year later, another survey found that 49% of adults who are unemployed and looking for a new job report to be pessimistic about their possibility to find one in the near future [2].

As far as the US situation is concerned, about half of non-retired adults (51%) say the economic impact of the coronavirus outbreak will make achieving their long-term financial goals harder [3]. The situation in developing countries is even worse, given that, according to a recent WHO estimate [4]. The Covid-19 pandemic is still causing the disruption of essential health services in 90% of the countries after one year. The crisis, in a nutshell, is having far-reaching and long-lasting implications for almost all aspects of the life of millions of people worldwide, and the increasing awareness among the general public of the magnitude and duration of its many effects is producing widespread emotional distress.

It is well known that our affective states, both positive and negative, have important behavioral consequences, strongly influencing our cognitive processes and deliberation abilities [5]. Therefore, in times of turbulence such as the present one, in which our daily habits and routines have been disrupted by radical limitations to sociality and mobility, and the future is highly uncertain, it is plausible to assume that people’s peculiar emotional reactions, besides affecting their well-being, might also significantly affect their decision-making outcomes and processes in a significant way. In this study we focus specifically on how negative emotions may affect two variables that influence our decision-making activity by specifically impacting the extent to which we are willing to sacrifice present gratifications for future ones, i.e., our “time preferences” (or temporal discounting), and the extent to which we are willing to take risks, i.e., our “risk attitudes” (or probability discounting).

The importance of studying temporal and probability discounting comes from the observation that both variables have a remarkable influence on decisions involving important aspects of our lives (e.g., savings [6], consumer decisions [7,8], employment [9,10], educational investment [11], energy conservation [12], and financial decisions [13]), many of which bear long term consequences, for example, suboptimal behaviors (e.g., health behaviors [14,15], obesity [16], smoke [17,18]) and clinical conditions (e.g., drug [19,20] and alcohol [21,22] abuse, and gambling disorder [23,24]).

We chose to test the impact of the entire range of the primary negative emotions (Primary or basic emotions are hypothesized to be a special class of emotions out of which all other emotions are compounded. According to most theorists, they are innate, universal, and distinct affective states which evolved to serve adaptive functions [4,25]) as they were all, to some extent, experienced by a relevant portion of the population during the pandemic. Further, we included an additional negative emotion to this spectrum, moral disgust: although moral disgust does not belong to the core set of primary emotions, we believe it is important to include it in our study, as many people may experience such emotion toward members of their community refusing to contribute to ending the pandemic (by, for example, refusing to wear a mask and/or refusing to get vaccinated).

In a nutshell, the results of our study showed that the elicitation of all emotions significantly made subjects more “impatient” and more risk-prone in comparison to a “neutral emotion” condition taken as baseline. In both tasks, all negative emotions were associated with the same direction of the effect, although differences were observed in their magnitude, especially for sadness. These results are at odds with the motivation appraisal theory [26,27] therefore we propose alternative explanations based on self-regulation theories.

These findings contribute to the literature on emotions and their effect on high-level cognitive functions, by directly comparing a wide range of negative emotions and partially explaining the rather inconsistent results observed in the previous literature.

Furthermore, they also contribute to a broader literature on economic decision making—which has traditionally been focused on the emotional arousal associated with intertemporal or risky decisions [28,29,30], by showing the effect of prior emotions on decision-making abilities, which are often understudied.

Finally, they might help shed some light on the psychological drivers of many choices that individuals made during and immediately after the pandemic.

The rest of the paper proceeds as follows: the following section defines each target emotion precisely and illustrates previous research on emotions and decision making, with particular attention to intertemporal choices and choices under risk. We also briefly review the current neuroscientific evidence about the main drivers of risk propensity and time discounting. The next section defines the hypothesis of the current study. A method and Results section follows, describing in detail the experimental design and the main results, separately for experiment 1 and experiment 2. The last section offers some concluding remarks and directions for future research.

### 1.1. Negative Emotions and Decision Making

After several months into the COVID-19 pandemic, many of us are still suffering the consequences of the emotional toll that the spread of the virus brings about. We might have experienced physical disgust due to contamination threats arising from even the simplest daily activities (like going to the supermarket), moral disgust because we have been (and still are, depending on the peculiar situation of each country and region) constantly exposed to behaviors that put us at risk, such as violating social distancing, refusing to wear a mask, or refusing to vaccinate; anger because of restrictions that limit our sociality or, alternatively, lack of restrictions that expose us to the risk of infection, fear of contracting the disease and facing its immediate and long term consequences, and sadness because of isolation, loneliness, and, in the worst cases, loss of dear ones. Finally, in a non-negligible portion of the population, we must add the fear of vaccines and their potential side effects.

In the present research, we aim to compare whether and to what extent the above-mentioned negative emotions might affect our time and risk preferences in decision making compared to a control condition. Hence, we provide a short review for each emotion with particular attention to their possible effect on risk and time discounting.

*Disgust* is defined as a feeling of revulsion, sometimes accompanied by nausea, along with a strong desire to withdraw from the eliciting stimulus [31]. From an evolutionary perspective, disgust may function primarily with the objective of rejecting offensive food [32,33,34] or, more generically, to avoid contact with substances that transmit pathogens [35]. The psychological and economic literature focused on the relationship between physical disgust and risk sensitivity: for example, it has been found a positive association between disgust and risk-aversion [36], similarly, another study demonstrated that disgust sensitivity is correlated with heightened risk perception [37]. More recently, a study found that disgust only dampens participants’ propensity to take the gain, but not the loss [38]. With respect to intertemporal decision making, the effects of disgust are understudied and mostly unknown. To the best of our knowledge, the only study investigating the effect of disgust on temporal discounting indicated that disgust sensitivity predicted a preference for larger later rewards [39].

Moreover, moral transgressions can trigger disgust responses [40,41,42,43], implying that situations involving a social threat may equally lead to efforts to protect oneself from more indirect potential sources of harm or to preserve social order [35]. Hence, disgust elicited by abstract socio-moral transgressions can be defined as moral disgust [44]. To the best of the authors’ knowledge, there is no previous research that systematically tested the relationship between moral disgust and risk-taking or intertemporal decision making. However, moral transgressions can elicit physical disgust responses, and therefore rejections or avoidance responses [45]: hence, it is plausible to expect that morally disgusted individuals will be more risk avoidant and more inclined to choose larger-later rewards. Conversely, other studies juxtapose moral disgust to anger, identifying differences between elicitors of disgust and anger in moral contexts, with disgust responding more to bodily-moral violations such as incest, and anger responding more to socio-moral violations such as theft [46]. However, the research so far does not allow to sharply distinguish between moral disgust, physical disgust, and anger. As a consequence, related predictions about the relationship between moral disgust and decision-making are difficult.

*Anger* is generally defined as a feeling of displeasure and hostility that arises after being exposed to any form of injustice or offense [47]. Unlike other negative emotions, anger has been associated with motivational approach tendency [48] which provides the impulse to “go toward” (i.e., approaching behavior; [49]), as opposed to the avoidance system, which provides the motivational impulse to “go away”. The link between trait anger and approach motivation is particularly relevant in risk-taking. In fact, angry people tend to have optimistic risk assessments [50] whereas other studies found that loss aversion is negatively influenced by anger [51].

The effects of anger on intertemporal decisions are not well investigated, as only a few studies can offer some indications of the possible relationship between anger and temporal discounting. Specifically, one study has investigated the interaction between state and trait anger, showing that people low on trait anger preferred large delayed rewards over smaller-immediate ones when in an angry mood, while people with high trait anger, when in an angry mood, showed an inverse preference (i.e., preferring small-immediate reward over a large delayed one; [52,53]). Another study, conducted on adolescents with conduct disorder showed a significant relationship between irritability and an increased preference toward immediate rewards [54].

*Fear* can be defined as the feeling of agitation and anxiety caused by perceptions of uncertainty in the environment, or by the presence of a physical danger [27]. Fear responses are coherent with its theorized evolutionary function of avoiding/escaping danger [55], as the main objective of fear is defending an organism from an imminent threat [56,57,58]. Both precautionary and self-protective motivations [59,60] characterizing fear are well documented and established in the risk-taking domain: in fact, fear was consistently found to trigger pessimistic judgments and risk-averse behaviors [61,62,63,64,65,66,67]. However, according to the evidence of increased risk-taking as a result of excitement [62,68], it was argued that depending on the characteristics of the task, fear can be reinterpreted as a state of excitement and consequently promote, rather than discourage, risk-taking [69]. Hence, the role of fear in risky choices is still open to debate. No study, to the best of our knowledge, has investigated the effect of fear on intertemporal decision making.

*Sadness* is a typical response to loss [26] that predicts disengagement from goals that can no longer be attended to [70,71], and it is characterized by avoidance motivation [72]. Sadness may also sometimes be described as a psychological pain accompanied by additional feelings of loneliness, distress, depression, anxiety, grief [72,73,74], and anguish [75]. Previous research has compared anger and sadness showing that sadness attenuates the unrealistic illusion of control typical of anger [50,76], generating higher risk aversion [77,78]. However, it was found that when the typical rumination response of sadness is prevented, subjects, although still sad, are less risk averse [79]. Hence, the evidence is still largely inconclusive.

A study conducted on a population of cigarette smokers has shown that, with respect to intertemporal decision making, sadness has the effect of increasing impatient choices for cigarette puffs, as a measure of delay discounting preference [80]. Such an effect appeared to be mediated by an increased self-focus of attention which, in turn, is induced by the feeling of sadness [81]. Along similar lines, it has been suggested that sadness increases impatience and creates a myopic focus on obtaining money immediately instead of later, a phenomenon known as myopic misery [82].

### 1.2. Hypothesis

Based on the literature reviewed above, our hypothesis is that all target emotions affect temporal discounting and risk propensity significantly with respect to the neutral condition. As for the direction of the effect, in accordance with the literature on the motivation appraisal of emotion, we expect the emotions associated with avoidance motivation and withdrawing behavior—such as disgust, fear, and sadness—to be associated with increased risk aversion and preference for delayed gratification, while emotions associated with approaching motivations, such as anger, to increase risk taking and promote impulsivity in intertemporal choices. As for moral disgust, according to the previous literature, it is not entirely clear whether this emotional response falls within the approaching or withdrawing motivational system, thus, precise predictions are difficult to formulate.

However, the approaching/withdrawing motivation theory of emotions, albeit compelling, may not be as straightforward as it seems for some emotions. For example, fear—which is usually described as a withdrawing/avoidance emotion (i.e., flight response)—can actually trigger also responses of freezing or fight, depending on the cognitive appraisal of the situation (e.g., based on the course of action that is more likely to defend the individual from the threat that activated the emotional response of fear). Moreover, there are additional appraisal components that might affect the proposed negative emotions, as for example a feeling of certainty and a sense of personal control over the (threatening) situation [83]. Based on these lines of reasoning and on the inconsistency of the previous results in the relevant literature, it is possible to observe different effects on decision making, as a result of different appraisals triggered by the target emotions. Nonetheless, we expect that all the emotions will significantly modulate both time and probability discounting.

## 2. Methods and Results

### 2.1. Experiment 1

#### 2.1.1. Participants

A total of 321 participants took part in the study, after providing written informed consent in accordance with the 1964 Declaration of Helsinki and the APA ethical standards in the treatment of our human sample. Participants were informed of their right to discontinue participation at any time. Sixty-two participants had to be excluded from the study because they did not show any variability in responses (i.e., participants selected either the immediate or the delayed option across all the 168 trials of the decision task), precluding the estimation of the decision indices. Hence, a total number of 259 participants (177 F; M_age_: 34.09 ± 11.12 s.d.) constituted the final sample. The sample size was determined based on a power analysis (G-Power; [84]); aiming at a medium to large effect size (f range = 0.25–0.40) with an α error probability of 0.05 and a power (1-β error probability) of 0.95, the required sample size was calculated between 130 and 210 participants.

Participants were recruited via the online platform Prolific.co, while the data collection was carried out via Qualtrics. Participants were recruited from all over the world and were required to speak English as a first language to participate in the study, in order to ensure a good comprehension of the experiment (see Section 2.2.1 for details). In particular, the sample was constituted by 191 participants from the United Kingdom (74%), 21 from South Africa (8%), 14 from Canada (5%) 9 from the USA (3%), and the remaining participants from other countries (9%; Ireland: 7; Australia: 5; New Zealand: 3; Poland: 3; Portugal: 3; India: 1; Greece: 1; Italy: 1). Participants received a fee for their participation in the study. All subjects gave written informed consent and were informed of their right to discontinue participation at any time.

Participants were randomly assigned to one of six possible experimental conditions: neutral (N = 46; 33 F; M_age_ = 35.39 ± 10.65 s.d.), moral disgust (N = 45, 33 F; M_age_ = 33.49 ± 11.42 s.d.) physical disgust (N = 42, 27 F; M_age_ = 34.24 ± 9.45), anger (N = 41, 24 F; M_age_ = 32.66 ± 12.05 s.d.), sadness (N = 39; 23 F; M_age_ = 32.23 ± 10.80) and fear (N = 46; 37 F; M_age_ = 36.11 ± 11.64).

#### 2.1.2. Tasks and Procedure

At the beginning of the experiment, participants were randomly assigned to one of the six experimental conditions (neutral, moral disgust, physical disgust, sadness, fear, and anger). In the first part of the experiment, participants were asked to read a short script (i.e., scenario) which was designed to prompt a specific target emotion, according to the experimental condition.

The scripts contained a description of one of the following scenes: (1) a train trip to visit a friend (neutral script; adapted from [85]) (2) an incestuous act between brother and sister (moral disgust script; adapted from [86]); (3) an old man who is vomiting (physical disgust script; adapted from [85]); (4) the death of a close relative (sadness script; adapted from [87]); (5) a car accident (fear script; adapted from [88]); (6) an unfair evaluation in a class assignment (anger script; adapted from [87]).

Right after the emotional script administration, participants were asked to complete an intertemporal choice task (ITC). During the task participants were asked to choose between an immediately fixed amount of money (i.e., GBP 10) and a delayed amount; the latter was parametrically varied across seven amounts (i.e., GBP 15, 25, 30, 40, 45, 55, and 60) and six waiting times (i.e., 7, 15, 30, 60, 90, and 180 days), thus obtaining 42 different choice pairs. Each choice pair was repeated four times, thus, the task included a total amount of 168 trials, which were randomly distributed across the experimental block [see [23,89,90,91,92,93] for a similar task design). Choices were hypothetical.

**Figure 1:**
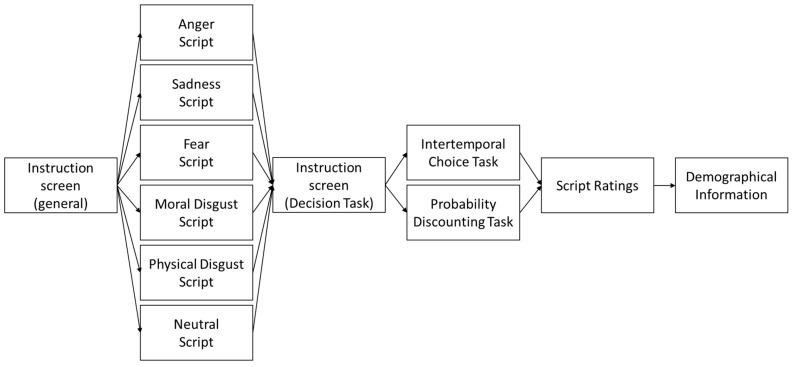
Experimental Procedure of Experiment 1 and Experiment 2. Participants were given some general information with respect to the structure of the experiment, then they were asked to read one of the six possible scenarios (randomly assigned). In the next phase, before taking part in the decision task, participants received the instruction (i.e., ITC in Experiment 1 and PD in Experiment 2). Finally, participants were asked to evaluate the story they read at the beginning of the experiment, according to the different emotions (i.e., anger, fear, sadness, moral and physical disgust), and were asked to provide some demographical information.

At the end of the ITC task, participants were asked to rethink about the script previously read and to rate the extent to which they experienced the specific target emotions (i.e., anger, fear, moral disgust, and physical disgust) on a 9-point Linkert scale ranging from 1 (i.e., not at all) to 9 (i.e., very much). The experiment ended with the collection of demographic information (Figure 1; see Appendix A for details).

#### 2.1.3. Data Analyses

As a first step, we conducted analyses aimed at verifying that the scripts elicited the target emotions. Thus, a linear mixed-effect model (LMM) was conducted using the emotional ratings as a dependent variable and emotion (i.e., Anger, Sadness, Fear, Moral Disgust, Physical Disgust) and condition (i.e., Neutral, Anger, Sadness, Fear, Moral Disgust and Physical Disgust) as fixed-effects, along with their interaction effects.

As a second step, we extracted individual discount rates (k parameter) from intertemporal choices using a standard routine also employed in many previous studies [90,94,95,96,97]. First, for each time delay, we calculated the fraction of times in which participants selected the future reward over the immediate one as a function of the objective amount of the delayed option. Then, the points of subjective equivalence (*pse*)—defined as the amount at which participant would choose the immediate and the delayed reward with equal frequency (i.e., 50%)—were estimated by fitting these data with a logistic function by employing an in-house MATLAB algorithm [98]. Subjective values (SV) for each time delay were thus calculated as the ratio between the immediate amount (GBP 10) and the *pse*, using the following equation:

$$\mathrm{SV}=\;\frac{10}{pse}$$

where 10 was the amount of money that was immediately available (i.e., GBP10). Finally, subject-specific discount rates (*k*) were estimated by fitting SV and time delays (*D*) with the hyperbolic function (using the MATLAB *cftool* [98]):

$$\mathrm{SV}=\frac{1}{1+kD}$$

The goodness-of-fit between the hyperbolic model and the data was overall high: R^2^ = 0.83. A logarithmic transformation, prior to statistical testing, was performed to account for skewed distribution (Kolmogorov–Smirnov test of normality: *p* > 0.20).

Finally, we entered the individual discount rates (log k) as the dependent variable in a liner mixed-effect model in order to test the specific effect of the target emotions on participants’ choice preferences. To this aim, an LMM was run using the fixed effect of the *condition* (i.e., Neutral, Anger, Fear, Moral Disgust, and Physical Disgust).

LMMs (both on individual discount rates and emotion ratings) were run employing the software R Studio [99] (lme4 and lmerTest packages [100,101,102]) and using a fully specified random effect structure by using the subjects, trials, age, and gender as random effects along with the random slopes of the fixed effects. Post hoc analyses were conducted using a false-discovery rate (FDR) correction for multiple comparisons [103,104,105]. All analyses were run using a 0.05 threshold for statistical significance.

The following section describes the main results of the three sets of analyses conducted.

#### 2.1.4. Results

Descriptive statistics of the emotional ratings (means ± standard deviations) across the six experimental conditions are reported in Table 1.

The first analysis was aimed at testing whether the scripts were able to elicit the target emotion that we intended to manipulate (see Table 2 for the detailed results).

**Figure 2:**
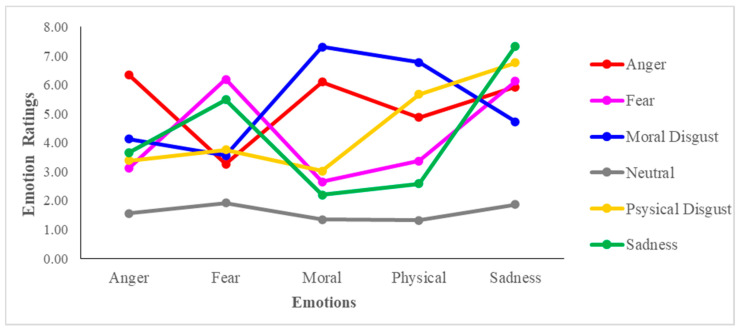
Results of the LMM model (Experiment 1). Analyses conducted on the individual ratings using the fixed effect ratings using the fixed effects of the condition (Ager, Sadness, Fear, Moral disgust, Physical Disgust, Neutral), the emotion (Anger, Sadness, Fear, Moral disgust, Physical Disgust), and their interaction effect.

Thus, to this aim, an LMM was run on the emotional ratings using the fixed effects of emotion (i.e., Anger, Sadness, Fear, Moral Disgust, Physical Disgust), condition (i.e., Neutral, Anger, Sadness, Fear, Moral Disgust and Physical Disgust) and their interaction term. The results revealed that the effect of emotion (*X^2^* = 4.43, *p* = 0.35) was non-significant, while the effect of condition (*X^2^* = 155.03, *p* < 0.001) was found significant. More importantly for the purposes of this analysis, the emotion by condition interaction was found significant (*X^2^* = 701.76, *p* < 0.001), indicating that all the scripts were eliciting the target emotions. Indeed, post hoc inspections revealed that in the Anger condition (Figure 2, red line), participants rated the anger emotion higher compared to Fear (*p* < 0.01) and Physical Disgust (*p* < 0.05), but it did not differ significantly from Moral Disgust (*p* = 0.86) nor Sadness (*p* = 0.76) In the Moral Disgust condition, the emotion of Moral Disgust (Figure 2, blue line) was rated higher compared to Physical Disgust (*p* < 0.05), Fear (*p* < 0.001), Sadness (*p* < 0.05) and Anger (*p* < 0.01). In the Physical Disgust condition (Figure 2, yellow line), participants rated Physical Disgust higher compared to Moral Disgust, Fear and Anger (all *p* < 0.05), but it did not differ from Sadness (*p* = 0.37). In the Fear condition (Figure 2, pink line), the emotion of Fear was rated higher compared to Anger, Moral Disgust and Physical Disgust (all *p* < 0.001), but not from Sadness (*p* = 0.97). Finally, all the target emotions were rated as very low in the Neutral condition, and no differences were observed among their ratings (all *p* > 0.05).

The results confirmed that the scripts were able to elicit the target emotions, nevertheless, some scripts also elicited some associated negative emotions (especially Sadness).

Thus, once established that the scripts elicited the target emotions, we conducted an additional analysis to test whether such emotions affected intertemporal choice patterns. Descriptive statistics (means and standard deviations) of the individual discount rates across the experimental conditions are displayed in Table 3.

**Figure 3:**
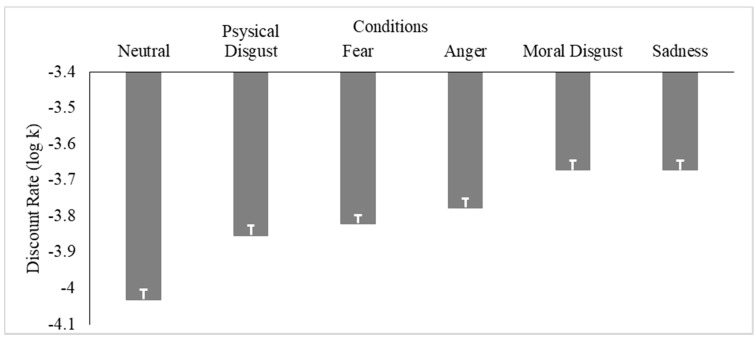
Results of the LMM (Experiment 1). Analyses conducted on the individual discount rates (log k) using the fixed effect of the condition (Anger, Sadness, Fear, Moral Disgust, Physical Disgust, Neutral).

An LMM was run on individual discount rates (log k) using the fixed effect of the condition (i.e., Neutral, Anger, Sadness, Fear, Moral Disgust, and Physical Disgust). The results revealed that the model yielded a statistically significant effect of the condition (X^2^ = 405.42, *p* < 0.001), indicating that the different target emotions elicited using the scripts did differentially affect intertemporal preferences in the choice patterns (see Figure 3, Table 4). Specifically, post hoc inspections revealed that when experiencing Anger, Sadness, Fear, Physical Disgust, or Moral Disgust, as compared to the neutral condition in which these emotions were not experienced, participants displayed a higher tendency toward preferring smaller-sooner rewards over larger-delayed ones (all *p* < 0.01); in other words, they discounted the future more steeply compared to the neutral condition. This tendency was more pronounced when experiencing Sadness and Moral Disgust—which did not differ statistically from one another (*p* = 0.99) but were both significantly higher compared to the other conditions (all *p* < 0.01). The effect was less pronounced on Fear and Anger, whose difference was not significant (*p* > 0.05), while they both differed from the Physical Disgust condition (*p* < 0.01), which was the emotion yielding the less marked effect.

### 2.2. Experiment 2

#### 2.2.1. Participants

A total of 258 participants took part in the study, after providing written informed consent in accordance with the 1964 Declaration of Helsinki and the APA ethical standards in the treatment of our human sample. Eighteen participants had to be excluded from the study because they did not show any variability in responses (i.e., participants selected either the certain or the uncertain option across all the 168 trials of the decision task), precluding the estimation of the decision indices. Hence, a total number of 240 participants (157 F; M_age_: 32.93 ±10.70 s.d.) constituted the final sample. The calculation of the sample size was the same as Experiment 1, as well as participants recruitment and task administration. The sample constituted 184 participants from the United Kingdom (77%), 18 from Africa (8%), 9 from Canada (4%), 8 from the USA (3%), and the remaining participants from other countries (6%; New Zealand: 2; China: 2; Portugal: 1; Poland: 1; Czech Republic: 1; Italy: 1; Greece: 1; Germany:1). Participants received a fee for their participation in the study. All subjects gave written informed consent and were informed of their right to discontinue participation at any time.

Participants were randomly assigned to one of six possible experimental conditions: neutral (N = 39; 26 F; M_age_ = 33.74 ± 9.84 s.d.), moral disgust (N = 36, 24 F; M_age_ = 32.97 ± 8.86 s.d.) physical disgust (N = 37, 27 F; M_age_ = 32.08 ± 9.24), anger (N = 37, 25 F; M_age_ = 32.65 ± 13.00 s.d.), sadness (N = 50; M_age_ = 32.04 ± 10.61 s.d.) and fear (N = 41; 29 F; M_age_ = 34.20 ± 11.79 s.d.).

#### 2.2.2. Tasks and Procedure

The structure of the experiment was the same as Experiment 1 (see Section 2.1. for details): first, we administered the emotional scenarios, then participants took part in a probability discounting task, then they rated the emotions elicited by the scenarios, and finally were required to provide demographical information. The same scripts of Experiment 1 were used to prompt a specific target emotion, according to the experimental condition.

Right after the emotional script administration, participants were asked to complete a probability discounting task (PD). During the task participants were asked to choose between an immediate certain amount of money (i.e., GBP 10) and an uncertain amount; this latter was parametrically varied across seven amounts (i.e., GBP 18, 30, 36, 48, 54, 66, and 72) and six probabilities (i.e., 10, 25, 40, 50, 70 and 90%), thus obtaining 42 different choice pairs. Each choice pair was repeated 4 times; thus, the task included a total amount of 168 trials, which were randomly distributed across the experimental block. Choices were hypothetical.

At the end of the PD task, participants were asked to rethink the script previously read and to rate the extent to which they experienced the specific target emotions (i.e., anger, sadness, fear, moral disgust, and physical disgust) on a 9-point Linkert scale ranging from 1 (i.e., not at all) to 9 (i.e., very much). The experiment ended with the collection of demographic information (Figure 1; see Appendix A for details).

#### 2.2.3. Data Analyses

As in Experiment 1, as a first step, we conducted analyses aimed at testing that the script elicited the target emotions. To this aim, a linear mixed-effect model (LMM) was conducted using the emotional ratings as a dependent variable and emotion (i.e., Anger, Sadness, Fear, Moral Disgust, Physical Disgust) and condition (i.e., Neutral, Anger, Sadness, Fear, Moral Disgust, and Physical Disgust) as fixed-effects, along with their interaction effects.

As a second step, we extracted individual probability discount rates (h parameter) from intertemporal choices using the same routine used in Experiment 1. First, for each probability, we calculated the fraction of times in which participants selected the uncertain reward over the certain one as a function of the objective amount of the uncertain option. Then, the points of subjective equivalence (*pse*)—defined as the amount at which participant would choose the certain and the uncertain reward with equal frequency (i.e., 50%)—were estimated by fitting these data with a logistic function using an in-house MATLAB algorithm [98]. Subjective values (SV) for each probability were thus calculated as the ratio between the immediate amount (GBP 10) and the *pse*, using the following equation:

$$\mathrm{SV}=\frac{10}{pse}$$

where 10 was the amount of money that was immediately available (i.e., GBP10). Finally, subject-specific probability discount rates (*h*) were estimated by fitting SV and the odds against winning θ [i.e., *(1 − p)/p; p* = probability of winning] with the following equation [106,107] (using the MATLAB cftool [98]):

$$\mathrm{SV}=\frac{1}{1+h\theta }$$

The goodness-of-fit between the model and the data was overall high: R^2^ = 0.88. A logarithmic transformation, prior to statistical testing, was performed to account for skewed distribution (Kolmogorov–Smirnov test of normality: *p* > 0.20).

Finally, a linear mixed-effect model was conducted on the individual probability discount rates (log h) in order to test the specific effect of the target emotions on participants’ choice preferences. The model was performed using the fixed effect of the condition (i.e., Neutral, Anger, Sadness, Fear, Moral Disgust, and Physical Disgust).

As in experiment 1, LMMs were run employing the software R Studio [99] (lme4 and lmerTest packages [100,101,102]) and using a fully specified random effect structure by using the *subjects*, *trials*, *age*, and *gender* as random effects along with the random slopes of the fixed effects. Post hoc analyses were conducted using a false-discovery rate (FDR) correction for multiple comparisons [103,104,105]. All analyses were run using a 0.05 threshold for statistical significance.

#### 2.2.4. Results

Descriptive statistics of the emotional ratings (means ± standard deviations) across the six experimental conditions are reported in Table 5.

As in Experiment 1, as a first step, an LMM was conducted to ensure that the scripts were able to elicit the intended emotions (see Table 6 for the detailed results).

**Figure 4:**
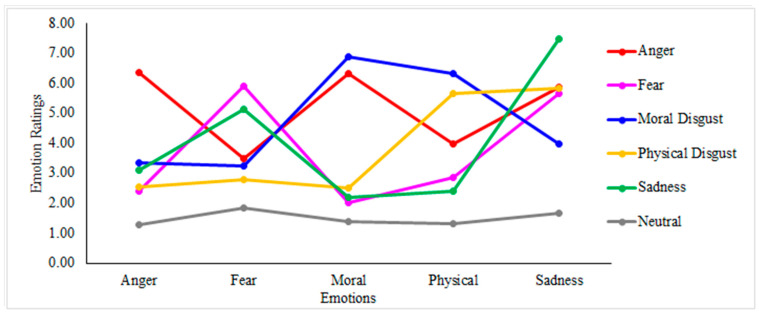
Results of the LMM model (Experiment 2). Analyses conducted on the individual ratings using the fixed effect ratings using the fixed effects of the condition (Ager, Sadness, Fear, Moral disgust, Physical Disgust, Neutral), the emotion (Anger, Moral disgust, Physical Disgust), and their interaction effect.

To this aim, the model was run using the individual ratings as a dependent variable and the fixed effects of the condition (Anger, Sadness, Fear, Moral Disgust, Physical Disgust, Neutral), the emotions (Anger, Sadness, Fear, Moral Disgust, Physical Disgust), and their interaction. The results showed the fixed effects of condition (*X^2^* = 140.51, *p* < 0.001) and emotion (*X^2^* = 226.11, *p* < 0.001) were both significant. More importantly for the purposes of the current analysis, the condition by emotion interaction was found significant (*X^2^* = 777.57, *p* < 0.001). The post hoc inspection showed that in the Anger condition (Figure 4, red line), participants rated the emotion of Anger higher compared to Physical Disgust (*p* < 0.001) and Fear (*p* < 0.001); nevertheless, the contrasts between Anger and Moral Disgust (*p* = 0.96) and Anger and Sadness (*p* = 0.24) failed to reach statistical significance. In the Fear condition (Figure 4, pink line), Fear was rated higher compared to all the other emotions (all *p* < 0.001) with the only exception of Sadness (*p* = 0.54). In the Moral Disgust condition (Figure 4, blue line), all comparisons were found statistically significant (Moral disgust—Anger: *p* < 0.001; Moral Disgust—Fear: *p* < 0.001; Moral disgust—Physical Disgust: *p* < 0.05; Moral Disgust—Sadness: *p* < 0.001), thus indicating that in this condition, Moral Disgust was felt more strongly compared to the other emotions. In the Physical Disgust condition (Figure 4, yellow line), Physical Disgust was rated higher compared to all the other emotions (*p* < 0.001), except for the Sadness (*p* = 0.66). In the Sadness condition (Figure 4, green line) the emotion of Sadness was rated higher compared to all the other emotions (all *p* < 0.001). Finally, in the Neutral condition (Figure 4, grey line) the three target emotions were all rated very low (~1.50), and no difference was observed among them (all *p* > 0.05).

Overall, the results confirmed that the scripts designed to elicit the target emotions were effective. Nevertheless, as in Experiment 1, Sadness was often activated along with the target emotion.

Secondly, we investigated whether the induction of the target emotions induced a modulation of probability choice behavior. Descriptive statistics (means and standard deviations) of the individual probability discount rates (log h) rates across the experimental conditions are displayed in Table 7.

**Figure 5:**
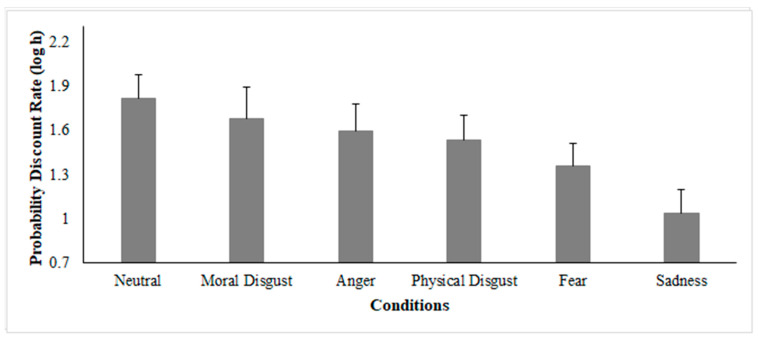
Results of the LMM (Experiment 2). Analyses conducted on the individual probability discount rates (log h) using the fixed effect of the condition (Anger, Sadness, Fear, Moral Disgust, Physical Disgust, Neutral).

To test this effect an LMM was run on the probability discount rates (log h) using the fixed effect of the condition (Anger, Sadness, Fear, Moral Disgust, Physical Disgust, Neutral). The results indicated that the fixed effect of the condition was statistically significant (*X^2^* = 1727.2, *p* < 0.01; Figure 5; Table 8). Specifically, the post hoc inspection revealed that, as compared to the neutral condition in which no specific emotion was induced, in the other five conditions it was possible to observe a reduction of the probability discount rates (all *p* < 0.01), thus indicating that such emotions had the effect of increasing individual tolerance of uncertain outcomes, thus, participants choose the uncertain (i.e., risky) option more often. Such effect was more pronounced in the Sadness condition and systematically decreased in the Fear, Physical Disgust, Anger, and Moral Disgust conditions, as all the post hoc comparisons were found statistically significant (*p* < 0.05).

## 3. Discussion

Across two experimental studies, we showed that when experiencing anger, sadness, fear, physical disgust, or moral disgust participants discount the future more steeply, i.e., they become significantly more impatient with respect to a neutral condition. This tendency is particularly pronounced in the case of sadness and moral disgust, and less pronounced for fear and anger, while physical disgust is the emotion yielding the less marked effect (Study 1). Secondly, anger, sadness, fear, physical disgust, and moral disgust generate a reduction of the probability discount rates, i.e., make individuals significantly more tolerant toward risk. The effect is more pronounced in the sadness condition and systematically decreases in the fear, physical disgust, anger, and moral disgust conditions (Study 2).

These results reverse the classical motivational appraisal approach according to which emotions associated with an approaching motivational appraisal, such as anger, should generate a higher risk propensity compared to the emotions associated with an avoidant motivational appraisal, as for example sadness, disgust, and fear [50].

However, when people feel an emotion, especially in the case of unpleasant or unwanted emotions—they often use specific emotion-regulation strategies in order to try to downregulate them [108]. The authors distinguish between two independent regulation strategies that can be differentially employed by individuals: antecedent-focused strategies versus response-focused strategies. The former are based on *cognitive reappraisal*, i.e., the reframing of a situation in order to change its emotional impact [109]. For example, one might reframe a reproach from one’s boss as an opportunity for personal growth, thus “transforming” a feeling of sadness into one of motivation. In contrast, response-focused strategies (i.e., expressive suppression) are based on the inhibition of the current emotion-expressive behaviors without acting upon the emotion itself [109], such as when hiding behavioral hints of anxiety during a job interview in order to make a good impression: in the latter case, the emotion is left unaltered, but its behavioral consequences are suppressed. These two strategies were studied by Heilman et al., (2010) in the context of decision making under risk and uncertainty, and the authors found that, especially for negative emotions, response-focused strategies did not impact upon risk-taking behavior, probably because these strategies do not change the underlying emotional experience, but only its behavioral manifestations. Conversely, response-focused strategies had the effect of increasing risky choices; according to the authors, because negative emotions are associated with risk avoidance, when people are able to successfully down-regulate the emotional experience, the effects of the negative emotion on risk-taking behavior are not observed anymore, i.e., people became more risk prone [110].

Nevertheless, an alternative explanation for these findings is that emotional reappraisal requires cognitive and attentional control, thus, “draining” cognitive resources from attending behavioral regulation in the risk choice paradigm. In other words, if attentional and cognitive resources are devoted to emotion reappraisal, participants may end up shorthanded in exerting control in other aspects of behavior [111,112]—such as deliberation and choice—as it is known that risk avoidance and intertemporal bargains require a certain degree of cognitive control [113]. Thus, a possible explanation of our results in the light of cognitive reappraisal theories may be that when feeling a negative emotion, participants may have tried to downregulate such emotion in order to feel better, thus diminishing the availability of cognitive control resources to devote to behavioral regulation.

A further and even more compelling explanation of our results derives from the observation that people tend spontaneously to engage in self-rewarding activities as a strategy aimed at down-regulating unpleasant emotions [114]. For example, it has been found that engaging in pleasant, rewarding activities is the most successful strategy for reducing negative affect [115]. Along these lines, a literature review has shown that in the remediation of negative mood/affect, self-reward represents an obvious and anecdotally frequent response, which also appears to be effective in producing a change or a reduction in the negative state [116,117].

According to this view, bad mood and negative emotions represent a frustration of the affective balance of an individual, who may react by using self-reward as a self-regulation strategy. Immediate rewards in intertemporal choice tasks and risky alternatives in probability discounting tasks represent forms of rewarding behavior. Indeed, it has been reliably shown that the preference for risk-taking behavior [118,119], as well as the preference for immediate rewards at the expenses of larger-later ones [96,120], is related to the activation of the mesolimbic reward system, i.e., a dopaminergic circuit responsible for our feeling of reward and pleasure [121]. According to this line of reasoning, the frustration of one’s emotional balance induced by a negative emotion should produce a tendency toward risky choices or immediate rewards, as a self-regulation strategy aimed at stimulating the mesolimbic reward system. Thus, the differences observed across the different emotions may be related to the extent to which a certain negative emotion threatens the individual’s emotional balance. Although the extent to which a specific negative emotion is considered as threatening for one’s emotional balance may be subject to individual differences [122], as well as to cultural expectations [123], we consistently found sadness to elicit the most pronounced effect across both experimental tasks. Sadness is often associated with a high degree of unacceptability, which is partially socially and culturally shaped. Cultural norms dictate that people are expected to strive for happiness and not to feel sad, as happiness has been enthusiastically promoted as important for personal well-being and a meaningful life [124]. Hence, people often struggle with the feeling of sadness and may be particularly motivated to down-regulate such emotion. In the context of the current study, this would determine a higher tendency toward the selection of immediate and risky options.

The interpretation of increased preference toward risky and impatient choices as an emotional regulation strategy of self-reward finds additional support also in other lines of literature. For example, studies on eating disorders, and in particular on binge-eating, have shown that dysregulated eating behaviors are used as a mechanism that suppresses or ameliorates the experience of negative affect [125,126,127], especially when the emotional state is experienced as unacceptable or wrong, thus determining the reliance on dysfunctional coping strategies [128].

Further support comes from some trends that have been observed during the Covid-19 outbreak. According to a study conducted by the Pew Research Center, in the third quarter of 2020, about 28.6 million Baby Boomers (People born between 1946 and 1964.) reported that they were out of the labor force due to retirement and that since February 2020, the number of retired Boomers has increased by about 1.1 million [129]. Although the mechanisms that led to this trend are yet to be tested, according to many financial advisors, the new attitude of “*life is too short*” is spreading among their customers, pushing them to take the plunge. Thus, after months of restrictions, regulations, bad mood, and sacrifices, the prospect of returning to the old pre-COVID working day appears no longer attractive, pushing people toward the prospect of a self-rewarding pre-retirement. The phenomenon could be explained both in the light of the “cognitive appraisal” interpretation by which negative emotions led to a reframing of one’s beliefs (“*a life of continuous work and sacrifice is not worth living*”) and in the light of the self-regulation strategy explanation (“*after months of distress I need a reward*”).

Similar considerations might apply also to another observed pandemic trend, i.e., the sharp increase in the homeownership rate in the US since the onset of the virus outbreak in February 2020. More specifically, the rate rose 1.2 percentage points for households headed by someone age 65 or older, and the increase from 2019 to 2020 was more substantial for households with family incomes below the national median [130]. Besides rational motivations (e.g., very low interest rates, foreclosure moratoriums, etc.) an “emotional” factor could have also played a role in pushing so many Americans to make such an important decision at this particular time.

### Limitations and Further Research

Given the massive exposure to a whole range of negative feelings generated by the COVID-19 pandemic, in this study we have systematically tested the effect of all primary negative emotions on two related and important determinants of economic choices: time and probability discounting. We also added moral disgust to the emotions investigated, although it is not considered a primary emotion, because we deemed it especially relevant in the context of the pandemic. Our results reverse the classical motivational appraisal approach, consistently identifying in sadness the negative emotion more likely to determine a higher tendency toward the selection of immediate and risky options. However, a recent study has shown steeper time discounting functions and higher risk propensity in depressed participants as compared to healthy ones [131], while an additional study showed no overarching tendency for the depressed to engage in either more or less risk-taking, and that differences in risk-taking behavior are largely explained by behavioral traits such as locus of control, optimism, and trust [132]. The same goes for the emotion of fear which has been traditionally associated with avoiding/withdrawing behaviors. Nevertheless, studies found that anxious participants show a higher tendency toward the selection on immediate vs. delayed choices [133], while others report no relationship between anxiety and risk preferences [134]. Hence, in general, the inconsistency of the results calls for further studies that investigate the specific mechanisms underlying the relationship between sadness and risk and shortsightedness propensity. Moreover, we studied negative emotions most likely elicited by the pandemic: however, in doing so, we used pre-validated scripts with no direct reference to the pandemic itself. Further research can corroborate our results using more context-specific scripts.

Finally, our study is the first that analyzed the effect of moral disgust on risk aversion and intertemporal choice; additional research is required in order to better delineate the role of this emotion in decision making, for example, comparing it not only with other negative emotions but also with positives ones.

A possible limitation of the current study is related to the use of emotional scripts which—in some cases—elicited more than a single emotion; on the one hand, eliciting multiple emotions can make it more difficult to disentangle the specific contribution of each emotion; on the other hand, it must be noted that in real life, emotions are rarely felt independently. Additionally, the employment of different methods to elicit emotions—such as the utilization of images or facial expressions—would be difficult to apply in the case of complex emotions such as moral disgust.

An additional limitation of the current study can be found in the use of hypothetical choices. Although several studies have reported no differences between hypothetical and real choices (involving actual monetary gain) in discounting tasks [135,136,137], further studies are necessary to corroborate our findings using a fully incentivized structure.

## 4. Conclusions

The current sanitary emergency has determined major changes in our lives and, in many cases, it has determined the unusually prolonged experience of a series of negative feelings. Hence, this study is aimed to investigate whether and to what extent negative feelings may impact decision-making capacities. In particular, we focused our investigation on time and probability discounting, because of their relevance and pervasiveness in many different domains of investigation. The results of this study suggest that anger, fear, sadness, moral disgust, and physical disgust have the effect of increasing the selection of immediate over delayed options—in intertemporal choice tasks—and risky over certain rewards—in a probability discounting task. While the direction of the effect was the same across all emotions, the magnitude of the effect appeared to be more pronounced for sadness and moral disgust. The current findings appear in line with theories of emotional regulation, and in particular, of self-reward. According to this view, experiencing a negative emotion impacts the subjective emotional balance, hence, triggering action necessary to restore such balance. In this view, the selection of immediate and risky rewards plays the role of self-reward by stimulating the dopaminergic mesolimbic system.

## Figures and Tables

**Table 1:** Means and standard deviation of the emotional ratings across the six experimental conditions (Experiment 1).

	Emotion Ratings				
	Anger	Fear	Moral Disgust	Physical Disgust	Sadness
Condition: Anger	6.34 ± 2.26	3.27 ± 2.50	6.10 ± 2.35	4.88 ± 2.39	5.93 ± 2.28
Condition: Fear	3.13 ± 2.28	6.20 ± 2.36	2.65 ± 2.21	3.37 ± 2.47	6.13 ± 2.42
Condition: Moral Disgust	4.13 ± 2.75	3.56 ± 2.89	7.31 ± 2.34	6.78 ± 2.62	4.73 ± 2.89
Condition: Physical Disgust	3.38 ± 1.21	3.76 ± 1.56	3.02 ± 0.94	5.67 ± 0.89	6.76 ± 1.50
Condition: Sadness	3.67 ± 2.22	5.49 ± 1.92	2.21 ± 2.19	2.59 ± 2.39	7.33 ± 1.81
Condition: Neutral	1.57 ± 2.73	1.91 ± 2.57	1.35 ± 1.70	1.33 ± 1.97	1.87 ± 1.86

**Table 2:** Results of the LMM, conducted in Experiment 1. Analyses conducted on the individual ratings using the fixed effects of the condition (Ager, Moral disgust, Physical Disgust, Neutral), the emotion (Anger, Sadness, Fear, Moral disgust, Physical Disgust), and their interaction effect.

	*β*	*S.E.*	*p*
Condition: Fear	−3.26	0.48	−6.82 ***
Condition: Moral Disgust	−2.27	0.48	−4.74 ***
Condition: Neutral	−4.78	0.48	−10.03 ***
Condition: Physical Disgust	−2.95	0.49	−6.02 ***
Condition: Sadness	−2.69	0.50	−5.41 ***
Emotion: Fear	−3.07	0.99	−3.10 **
Emotion: Moral Disgust	−0.24	0.99	−0.25
Emotion: Physical Disgust	−1.46	0.99	−1.48
Emotion: Sadness	−0.41	0.99	−0.42
Condition: Fear by Emotion: Fear	6.14	0.51	12.06 ***
Condition: Moral Disgust by Emotion: Fear	2.50	0.51	4.88 ***
Condition: Neutral by Emotion: Fear	3.42	0.51	6.72 ***
Condition: Physical Disgust by Emotion: Fear	3.45	0.52	6.64 ***
Condition: Sadness by Emotion: Fear	4.89	0.53	9.23 ***
Condition: Fear by Emotion: Moral	−0.23	0.51	−0.46
Condition: Moral Disgust by Emotion: Moral Disgust	3.42	0.51	6.69 ***
Condition: Neutral by Emotion Moral Disgust	0.03	0.51	0.05
Condition: Physical Disgust by Emotion: Moral Disgust	−0.11	0.52	−0.22
Condition: Sadness by Emotion: Moral Disgust	−1.22	0.53	−2.30 *
Condition: Fear by Emotion: Physical Disgust	1.70	0.51	3.34 ***
Condition: Moral Disgust by Emotion Physical Disgust	4.11	0.51	8.03 ***
Condition Neutral by Emotion: Physical Disgust	1.22	0.51	2.40 *
Condition: Physical Disgust by Emotion: Physical Disgust	3.75	0.52	7.20 ***
Condition: Sadness by Emotion: Physical Disgust	0.39	0.53	0.73
Condition: Fear by Emotion: Sadness	3.41	0.51	6.71 ***
Condition: Moral Disgust by Emotion: Sadness	1.01	0.51	1.98 *
Condition: Neutral by Emotion: Sadness	0.72	0.51	1.41
Condition: Physical Disgust by Emotion: Sadness	3.80	0.52	7.29 ***
Condition: Sadness by Emotion: Sadness	4.08	0.53	7.70 ***

* *p* < 0.05 ** *p* < 0.01; *** *p* < 0.001.

**Table 3:** Means and standard deviation of individual discount rates (log k) across the six experimental conditions (Experiment 1).

	Anger	Fear	Moral Disgust	Neutral	Physical Disgust	Sadness
Mean	−3.78	−3.82	−3.67	−4.03	−3.85	−3.67
Standard deviation	1.18	1.17	1.27	1.3	1.35	1.24

**Table 4:** Results of the LMM, conducted in Experiment 1. Analyses conducted on the individual discount rates (log k) using the fixed effects of the condition (Ager, Moral disgust, Physical Disgust, Neutral).

	*β*	*S.E.*	*p*
Condition: Fear	0.01	0.02	0.43
Condition: Moral Disgust	0.14	0.02	6.77 ***
Condition: Neutral	−0.22	0.02	−10.66 ***
Condition: Physical Disgust	−0.06	0.02	−3.02 **
Condition: Sadness	0.11	0.02	5.00 ***

** *p* < 0.01; *** *p* < 0.001.

**Table 5:** Means and standard deviation of the emotional ratings across the six experimental conditions (Experiment 2).

	Emotion Ratings				
	Anger	Fear	Moral Disgust	Physical Disgust	Sadness
Condition: Anger	6.35 ± 2.33	3.49 ± 2.07	6.32 ± 2.49	3.97 ± 2.34	5.86 ± 2.34
Condition: Fear	2.39 ± 1.65	5.90 ± 1.96	2.02 ± 1.62	2.88 ± 1.98	5.66 ± 2.07
Condition: Moral Disgust	3.36 ± 2.16	3.25 ± 2.36	6.89 ± 2.09	6.33 ± 2.47	4.00 ± 2.76
Condition: Physical Disgust	2.54 ± 0.96	2.78 ± 1.66	2.51 ± 1.12	5.65 ± 1.02	5.84 ± 1.16
Condition: Sadness	3.12 ± 2.15	5.14 ± 2.04	2.18 ± 1.97	2.40 ± 2.77	7.48 ± 2.60
Condition: Neutral	1.28 ± 2.22	1.85 ± 2.81	1.38 ± 1.72	1.33 ± 1.90	1.67 ± 1.80

**Table 6:** Results of the LMM, conducted in Experiment 2. Analyses conducted on the individual ratings using the fixed effects of the condition (Ager, Fear, Moral disgust, Physical Disgust, Neutral), the emotion (Anger, Sadness, Fear, Moral disgust, Physical Disgust), and their interaction effect.

	*β*	*S.E.*	*p*
Condition: Fear	−3.99	0.48	−8.35 ***
Condition: Moral Disgust	−2.99	0.49	−6.09 ***
Condition: Neutral	−5.10	0.48	−10.55 ***
Condition: Physical Disgust	−3.87	0.49	−7.92 ***
Condition: Sadness	−3.30	0.46	−7.24 ***
Emotion: Fear	−2.87	0.38	−7.49 ***
Emotion: Moral Disgust	−0.03	0.38	−0.07
Emotion: Physical Disgust	−2.38	0.38	−6.22 ***
Emotion: Sadness	−0.49	0.38	−1.27
Condition: Fear by Emotion: Fear	6.38	0.53	12.08 ***
Condition: Moral Disgust by Emotion: Fear	2.75	0.54	5.05 ***
Condition: Neutral by Emotion: Fear	3.43	0.53	6.42 ***
Condition: Physical Disgust by Emotion: Fear	3.11	0.54	5.74 ***
Condition: Sadness by Emotion: Fear	4.89	0.50	9.68 ***
Condition: Fear by Emotion: Moral Disgust	−0.34	0.53	−0.64
Condition: Moral Disgust by Emotion: Moral Disgust	3.56	0.54	6.52 ***
Condition: Neutral by Emotion: Moral Disgust	0.13	0.53	0.24
Condition: Physical Disgust by Emotion: Moral Disgust	0.00	0.54	0.00
Condition: Sadness by Emotion: Moral Disgust	−0.91	0.50	−1.81 ^†^
Condition: Fear by Emotion: Physical Disgust	2.87	0.53	5.43 ***
Condition: Moral Disgust by Emotion: Physical Disgust	5.35	0.54	9.82 ***
Condition: Neutral by Emotion: Physical Disgust	2.43	0.53	4.55 ***
Condition: Physical Disgust by Emotion: Physical Disgust	5.49	0.54	10.14 ***
Condition: Sadness by Emotion: Physical Disgust	1.66	0.50	3.29 **
Condition: Fear by Emotion: Sadness	3.76	0.53	7.11 ***
Condition: Moral Disgust by Emotion: Sadness	1.13	0.54	2.07 *
Condition: Neutral by Emotion: Sadness	0.87	0.53	1.63
Condition: Physical Disgust by Emotion: Sadness	3.78	0.54	6.99 ***
Condition: Sadness by Emotion: Sadness	4.85	0.50	9.60 ***

* *p* < 0.05; ** *p* < 0.01; *** *p* < 0.001; ^†^ *p* < 0.1.

**Table 7:** Means and standard deviation of individual probability discount rates (log h) across the six experimental conditions (Experiment 2).

	Anger	Fear	Moral Disgust	Neutral	Physical Disgust	Sadness
Mean	1.81	1.68	1.59	1.53	1.36	1.04
Standard deviation	1.1	1.41	1.25	1.13	1.04	1.09

**Table 8:** Results of the LMM, conducted in Experiment 2. Analyses conducted on the individual probability discount rates (log h) using the fixed effects of the condition (Ager, Moral disgust, Physical Disgust, Neutral).

	*β*	*S.E.*	*p*
Condition: Fear	−0.12	0.02	−5.49 ***
Condition: Moral Disgust	0.23	0.02	11.13 ***
Condition: Neutral	0.33	0.02	15.49 ***
Condition: Physical Disgust	−0.08	0.02	−3.89 ***
Condition: Sadness	−0.38	0.02	−18.76 ***

*** *p* < 0.001.

## Data Availability

Data are available at Mendeley Data, V1, doi:10.17632/x6y5vkttcf.1.

## Appendix A. Exmperiments’ Structure

The current experiment is composed by three sub-sections:

- In the first part, you will be asked to read a short story. Please, try to focus on the story and imagine it as vividly as possible.
- In the second part of the experiment, you will be asked to make a series of monetary choices. Beware that the task comprises a large amount of choices (>100) and that some choice options will be repeated more than once. Please, try to keep your attention up during the entire task.
- Finally, you will be asked to give some ratings, with respect to your feelings during the experiment.

Thank you for your participation!

Please read and imagine as vividly as possible the following story (each participant was randomly assigned to one of the six possible scenarios, hence each participant only read one story):

### Appendix A.1. Physical Disgust

You work as an assistant in home care. You open the door at an old man. You hear from the sounds coming from the chamber that something is wrong. You open the door. The man appears sick and has vomited, apparently, he was not able to reach the bathroom in time. You can feel the smell of vomit even from where you stand, it feels acre and sour. You walk up to the man; you can see that fresh vomit mixes with the dried-up remains of vomit from an hour ago. Observe the lumps of vomit that drip down along his chin. The man vomits again. The sound is nauseating you; you are gagging, you can feel your stomach tightening, and a sour taste in your mouth. While you help the man out of his clothing, you notice that he has also defecated himself. The smell is unbearable and nauseating. You would like to look aside, but you have to help him out. You feel the urge of vomiting yourself more and more, but you need to concentrate on your work.

### Appendix A.2. Moral Disgust

James and Holly are brother and sister. After they both graduate from college, they share an apartment in a large building with other flat mates. One night they end up alone in the apartment during a thunderstorm. There is a blackout. Holly is afraid and goes in her brothers’ room to find some consolation. James holds her to make her feel safe. Then, James and Holly start kissing on their mouth passionately, using their tongues. After some kissing, they eventually start touching each other’s genitals lustfully. The excitement grows until they end up having a sexual intercourse. They had sex many times that night. They used multiple forms of birth control, including a condom. Both enjoyed the sex very much, and the memory of that experience makes them feel closer. Nevertheless, they agreed to never speak about this with anyone, including each other, and to never do it again. This activity never creates any problem for either of them.

### Appendix A.3. Anger

You are enrolled in a course that is a prerequisite for your intended major. You are finding the course quite interesting; however, you do not get along with your T.A.: you often disagree with him/her, and he/she is highly critical and frequently scoffs at your comments. Recently, you wrote a big paper for the class that your T.A. graded. You were really interested in the paper topic and put a lot of effort into researching it. You believe this is one the best papers you had ever written. When the T.A. hands the papers back, you see that he/she has given you a “C-”, and during the next discussion section, he/she passes out copies of your paper as an example of a bad work. Although you are not mentioned by name, it is obvious by his/her frequent looking at you who wrote the paper. You decide to drop the class, despite knowing that it is offered once a year and will throw your fulfilling of your major’s requirements.

### Appendix A.4. Sadness

You are relaxing at home when you receive a call from your father. The minute you talk to him you know by his strained voice that something is wrong. He tells you that your mom is sick in the hospital, and that they don’t know what it is. Without finding out more you say you’ll go at the hospital immediately. In your way to the hospital constantly reassure yourself that your mom is OK and that it is nothing serious. Upon arrival you find your mom’s room: you see the rest of your family there with their pale drained faces and teary eyes. You go to your mom’s bed and kneel beside her. Her face rocks semi-consciously, flinching from time to time, and sometimes whimpering at the pain in her body. You reassure her that she’ll be all right, but she closes her eyes and tells you that she feels like she is spinning around. She then closes her eyes and dies. You can’t believe what is happening and you crouch over and hug your mom.

### Appendix A.5. Fear

On the way back to the city from a daytrip, you are driving on a winding mountain road. Right at the moment of making a turn, all of a sudden, you hear a great sound “Bomb!”, you feel that you’ve hit something severely and you begin to crash down the hill. Then you lose your consciousness. After you revive, you feel sticky all over and then you realize that it is all your blood. You are covered and surrounded by pieces of broken glasses. The car doors are twisted. Your legs are stuck in the car. You cannot move as it hurts so much. You feel the twists of pain all over and cannot see anything as well. It is completely dark inside the car; you only smell the mixed air of blood and mud. You do not know where you are nor how badly you are injured. Suddenly, the car shakes with great force, and you seem to hear branches breaking off. It seems that the car is hanging in the middle of the sky and will fall off at any moment, life or death seems to be a matter of fate...

### Appendix A.6. Neutral

You are about to look up a friend who has moved to another city. You are standing at the platform of the station, waiting for the right train, while the platform gradually becomes busier. The train in now approaching the platform. You are standing at the door, then you get in the train and start searching for a seat. Once you have found a seat, you lay down your stuff and sit down. Then, the conductor comes in and asks for the tickets. You look in your pockets and show it to the conductor. In some minutes, you will arrive at the station of your destination. The train decreases in speed, you have arrived. You take your case and get up. You are walking through the station in search of the exit, following the stream of people. Following the route description, you walk through the streets of the city and eventually reach the street and the house number where you must be.

The first part of the experiment is over.

In the second part you take part in a decision task, you which you will be asked to express your preference between two choice alternatives (Each participant took part only in one of the two decision tasks, hence only one description (i.e., intertemporal choice task vs. probability discounting task) was visualized by each participant):

Intertemporal choice task

- one of the alternatives is an amount of money immediately available (e.g., GBP 5 now),
- while the other is a larger amount of money available in a variable time interval (e.g., GBP 15 in 60 days).
- Probability discounting task
- one of the alternatives is an amount of money available for sure (e.g., GBP 5 at 100% certainty),
- while the other is a larger amount of money available at different levels of certainty (e.g., GBP 15 at 60% certainty).

Your task is to select the choice that you would prefer as realistically as possible, as if you could actually receive that amount of money.

There is no right or wrong choice, you only need to select the one that you would prefer. Additionally, you will need to consider each alternative independently from the others.

Beware that the task comprises a large amount of choices (>100) and that some choice options will be repeated more than once. Please, try to keep your attention up during the entire task.

Thank you!

Which alternative would you prefer? (Example of the structure of the trial).

- GBP 10 now/GBP 10 at 100% certainty
- GBP 40 in 30 days/GBP 40 at 30% certainty

The second part of the experiment is over.

In this third part, you are kindly asked to rate your emotions with respect to the story that you read at the beginning of the experiment. There is no right or wrong answer, just express how much you felt a specific emotion while reading and imagining the story.

Thank you!

Thinking about the story that you previously read, please indicate to which extent the story made you feel as …

	**1** **Not at All**	**2**	**3**	**4**	**5**	**6**	**7**	**8**	**9** **a Lot**
Angry	○	○	○	○	○	○	○	○	○
Morally Disgusted	○	○	○	○	○	○	○	○	○
Physically Disgusted	○	○	○	○	○	○	○	○	○
Sad	○	○	○	○	○	○	○	○	○
Fearful	○	○	○	○	○	○	○	○	○

Demographical Information:

- What is your age?
- What is your nationality?
- What is your gender?

Thank you for your participation!
